# Successful cervicothoracic esophageal stricture treatment with partial sternectomy and a pedicled TAAP flap: A case report

**DOI:** 10.3389/fsurg.2022.905241

**Published:** 2023-01-06

**Authors:** Baofei Li, Haiyang Wang, Jun Liu, Xiaosong Mu, Feng Xu, Di Deng, Yixin Qiao, Shipin Wang, Fei Chen

**Affiliations:** ^1^ Department of Otolaryngology, Head and Neck Surgery, West China Hospital, Sichuan University, Chengdu, China; ^2^ Department of Otolaryngology, Head and Neck Surgery, Langzhong People's Hospital, Langzhong, China

**Keywords:** esophageal stenosis, anastomotic fistula, sternectomy, esophagoplasty, perforator flap

## Abstract

Postoperative benign esophageal anastomotic leakage and stenosis are common complications after esophagectomy. Treatment options for anastomosis stenosis include endoscopic mechanical dilation, dilation-combined steroid injection, incisional therapy, stent placement, and self-bougienage. However, long-segmental cervicothoracic esophageal stenosis and cutaneous fistula are always refractory to conservative treatments and are clinically challenging. When lesions extend well below the thoracic inlet, transthoracic esophagectomy and alimentary canal reconstruction seem to be the common choice but are susceptible to perioperative mortality and donor-site sequelae, especially for patients with poor health conditions. In this report, we present a novel surgical approach for cervicothoracic esophageal stenosis and fistula *via* partial sternectomy and reconstruction with a pedicled thoracoacromial artery perforator flap. No recurrence or complications occurred throughout 3 months of follow-up. This case study adds new perspectives to the treatment of anastomotic stenosis.

## Introduction

Anastomotic stenosis is common in patients after esophagectomy. However, instances of long-segmental cervicothoracic esophageal stenosis and cutaneous fistula refractory to conservative treatments are rare. Treatment options for anastomosis stenosis include endoscopic mechanical dilation, dilation-combined steroid injection, incisional therapy, stent placement, and self-bougienage. Here, we report a case of a patient with a long-segmental cervicothoracic esophageal stricture that occurred several months after esophagectomy, which was successfully treated with sternectomy and a pedicled thoracoacromial artery perforator (TAAP) flap without transthoracic esophagectomy and alimentary canal reconstruction.

## Case presentation

**Figure 1:**
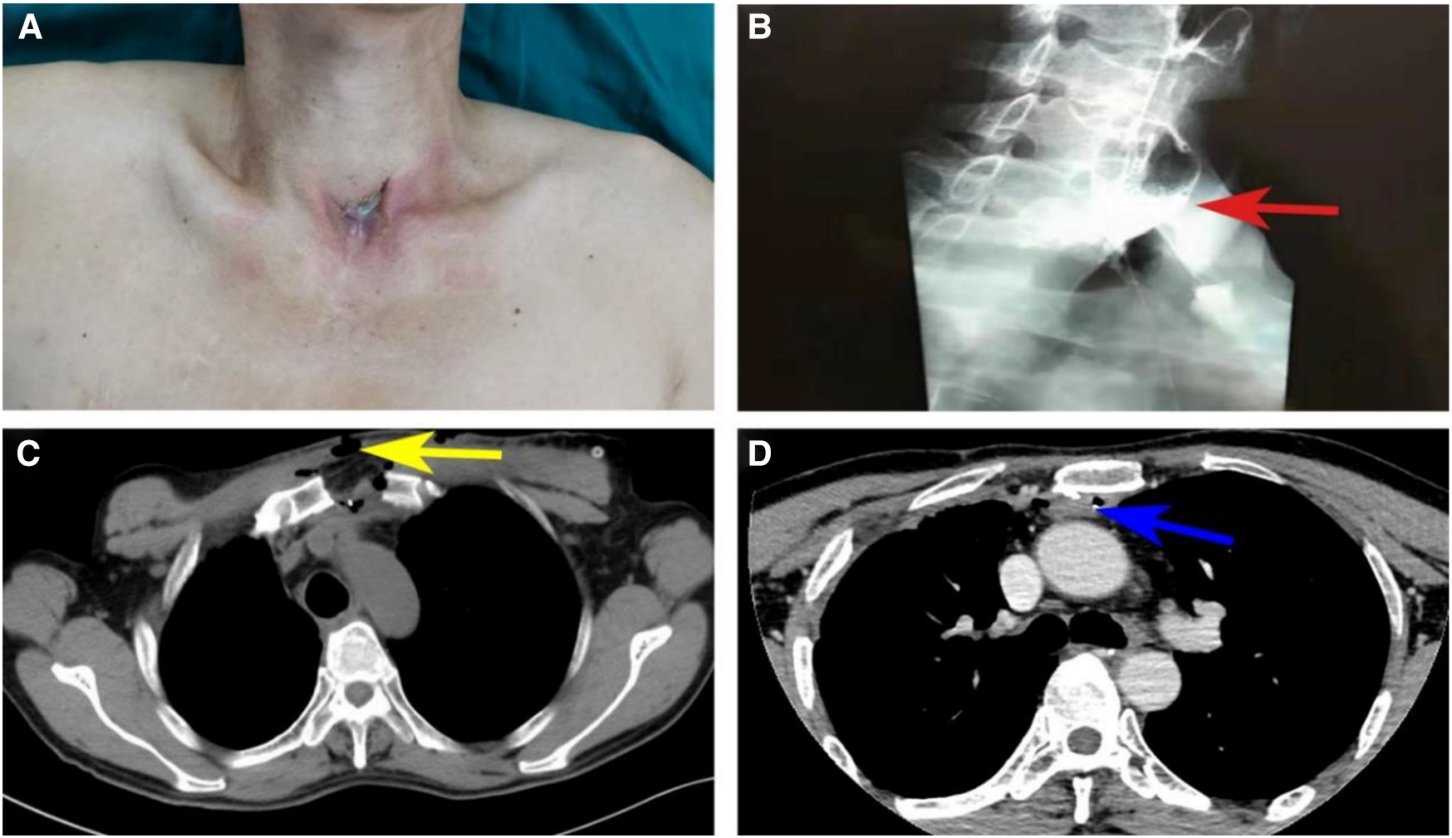
Preoperative visual inspection, esophagography and CT. (**A**) Visual inspection of external neck skin and esophageal cutaneous fistula; (**B**) Left lateral view from a barium swallow shows ingested barium extravasated from a perforation (red arrows) into extraluminal space with the lower part of the esophagus not visible, indicating esophageal stenosis and fistula. (**C**) CT shows the esophageal cutaneous fistula (yellow arrows). (**D**) CT shows the lower end of esophageal stricture (blue arrows) in T5.

A 52-year-old man with a known history of esophageal cancer had underwent esophagectomy and left cervical esophagogastrostomy *via* the retrosternal route in a different hospital 6 years prior to his presentation in our hospital. On the sixth postoperative day, the patient presented with a cervical anastomotic leak but was healed after adequate drainage, dressing changes, and the initiation of positive anti-infection measures. During the chemoradiotherapy treatment initiated 5 months after the operation, he developed esophageal anastomotic stenosis and refractory fistula. Tumor recurrence was prevented by performing endoscopic biopsy. The patient was subjected to multiple bougie dilations, along with twice fully covered metal stent placements, over the last 6 years. However, his dysphagia and intermittent cervical discharge did not disappear. Symptoms exacerbated in the last 2 months and the patient could be fed only with a nasogastric tube. He was referred to our department for further treatment. On physical examination, a cervical anastomotic fistula with gastric juice discharge was observed on the suprasternal fossa (Figure 1A). Esophagography in the other hospital revealed a stricture and leakage behind the sternum (Figure 1B). A contrast-enhanced CT scan in our hospital revealed an esophageal stricture extending from the level of C7 to T5 and a fistula between the esophageal lumen and skin (Figures 1C,D). Thus, the diagnosis concluded to cervicothoracic esophageal stenosis with a fistula after esophagectomy.

**Figure 2:**
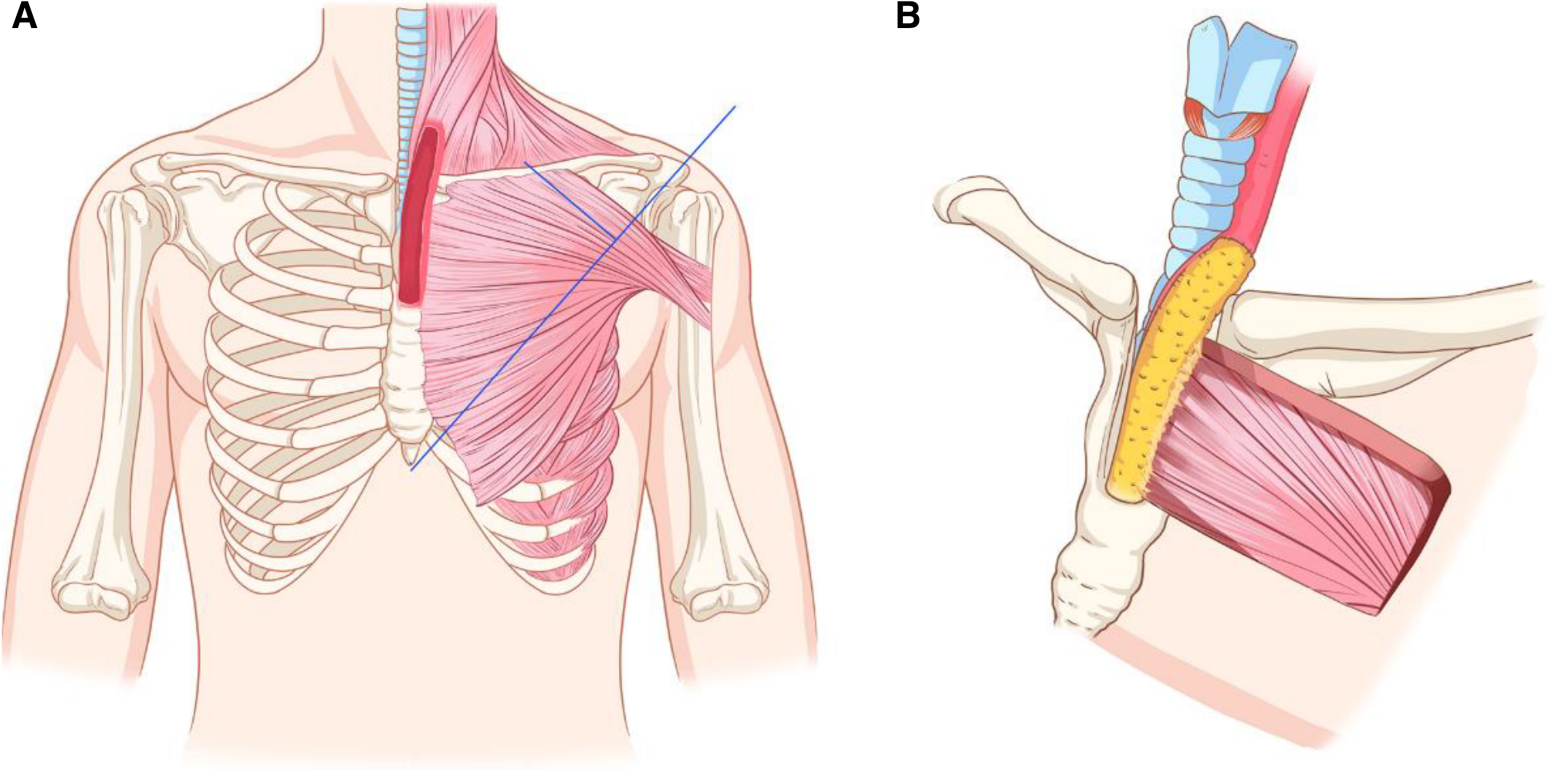
The preoperative Schematic diagram of designing of pedicled TAAP flap and the reconstruction process for this case. (**A**) Designing of a PTTA flap. (**B**) The flap was sutured to the residual esophagus.

We scheduled the transcervical approach combined with partial sternectomy to achieve complete lesion exposure, leaving open the possibility that cervicothoracic esophageal defects that occur after radical resection could be reconstructed with a thin and pliable pedicled flap. The pedicled TAAP flap has been widely used in hypopharyngeal and cervical trachea reconstruction. The flap size can be tailored up to 6 cm×12 cm, with a mean thickness of 0.5 cm (1). The vascular pedicle of the flap can be identified by an intersection of a line joined from the acromion to the xiphoid process and a perpendicular line drawn from the midclavicular point preoperatively (Figure 2).

**Figure 3:**
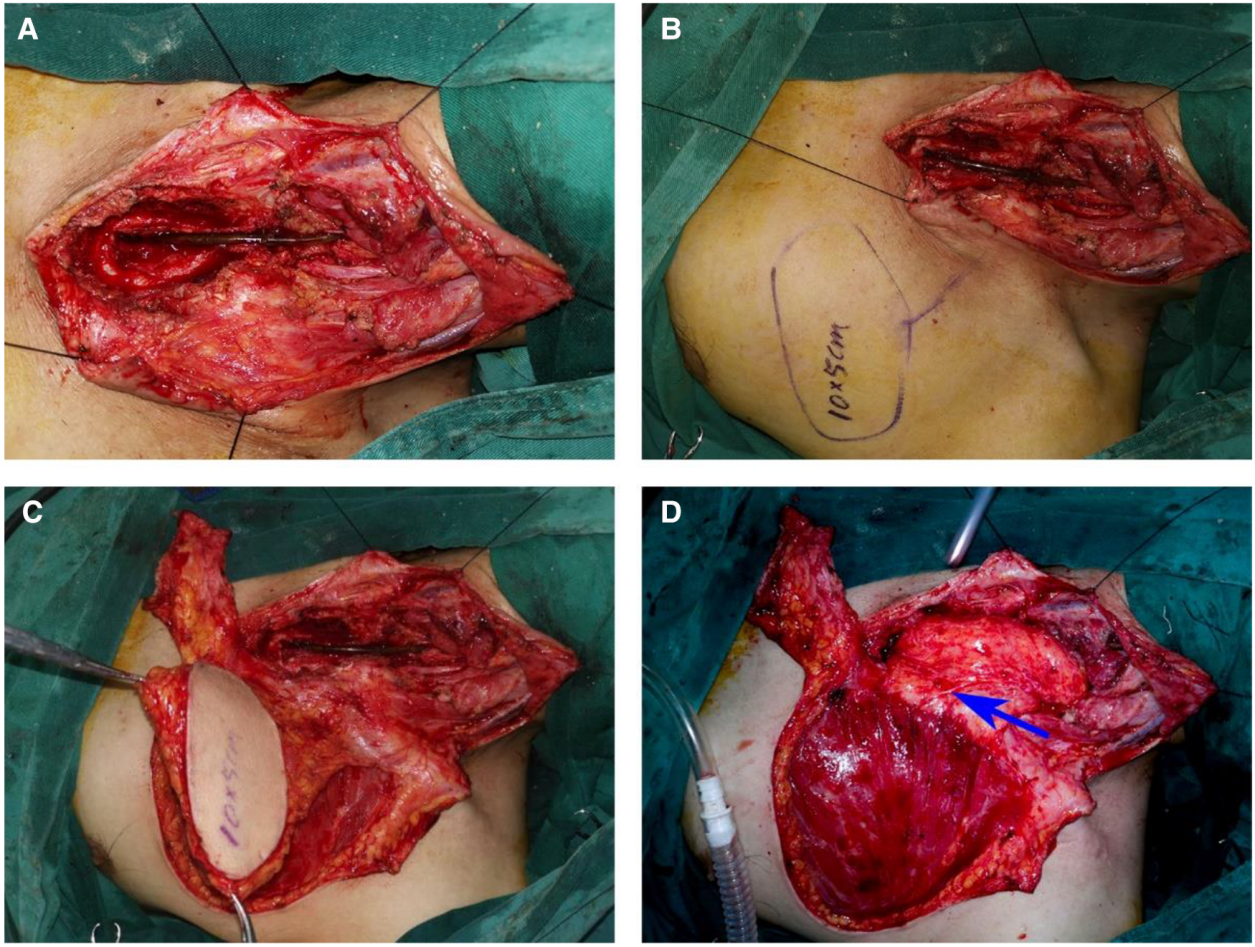
Stricture resection and reconstruction of the esophageal defect using a pedicled TAAP flap. (**A**) The defects of cervical and thoracic esophagus. (**B**) Design of a pedicled TAAP flap. (**C**) The harvesting of a pedicled TAAP flap. (**D**) Reconstruction of the defect of cervical and thoracic esophagus using a pedicled TAAP flap and thoraco-acromial artery (blue arrow)

Tracheal intubation was done and general anesthesia was administered. A T-shaped incision was made on the fistula to expose the cervical fistula and stenosis. Then, a part of the sternum adjacent to the first to third costal cartilage was resected to expose the upper thoracic esophageal stenosis. Intraoperative exploration revealed the length of stenosis to be approximately 8 cm. Cervical fistula and cervicothoracic esophageal stenosis were resected, while the posterior wall of the esophagus was retained. To reconstruct the near-circumferential esophageal defects, we designed and harvested a 10 cm×5 cm pedicled TAAP flap. The flap was contra-rotated 90°, rolled into a semitubular shape, and anastomosed to the distal stomach stump and proximal cervical esophageal remnant with a 3-0 (polyglactin 910, Ethicon) continuous suture. The wound of the donor site was closed directly (Figure 3). The total operative duration was approximately 5 h. From a pathological standpoint, no malignant cells were observed in the resected esophagus.

**Figure 4:**
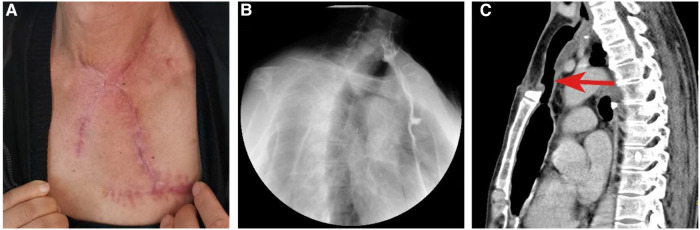
Postoperative visual inspection, esophagography and CT scan. (**A**) Postoperative visual inspection of external neck skin. (**B**) Esophagography shows the function of the reconstructed esophagus at the second month postoperatively. (**C**) CT shows the spacious reconstructed esophagus at the second month postoperatively.

Nasogastric nutrition and positive anti-infection measures were initiated on postoperative Day 1. The patient was discharged from the hospital on postoperative Day 14 with no complications, and no donor-site sequelae were observed. Despite partial sternum resection, the chest wall stabilized well, and the patient did not complain of dyspnea. Esophagography and CT 2 months after the operation revealed a viable anastomosis site with wide patency (Figure 4). A full liquid oral diet was resumed at a postoperative period of 2 months, while a solid oral diet was resumed at 3 months. A satisfactory recovery of swallowing function was obtained after a 3-month follow-up.

## Discussion

Anastomotic stenosis is common in patients after esophagectomy. Stenosis has been reported to occur in 5%–46% of patients (2). Patients suffer from significant dysphagia and many are tube-feed-dependent. Early strictures are usually related to ischemia at anastomosis, while later strictures are often associated with local tumor recurrence (3). Several studies have identified risk factors that predispose to stricture formation and have cited the following: cardiovascular diseases, intraoperative blood loss, poor vascularization of the gastric tube (in those procedures using this technique), the type of anastomosis (hand sewn vs. stapled, cervical vs. thoracic), and postoperative complications such as leakage, fistula formation, and infection at the anastomotic site (2, 4–7). In our case, the patient had undergone postoperative chemoradiotherapy and recurrent cutaneous anastomotic fistula, placing him at a high risk for stricture.

Several methods have been reported to treat esophageal stricture. The preferred treatment for benign esophageal strictures is endoscopic mechanical dilation including balloon dilation, Savary bougie dilation, and Eder-Puestow olive dilation (4, 7). Although most patients are effectively treated with up to five dilations, approximately 10% of patients need continuous dilations to achieve a dilation-free condition (8). In refractory strictures, intralesional steroid injection, combined with dilation therapy, esophageal stenting techniques, or incisional therapy, has been reported with variable success rates (4, 8). If the structures are still refractory, self-bougienage by using Maloney dilators may be a safe and effective alternative (4, 9). Finally, as previously described by Van Boeckel and Siersema, surgery should be a last-resort treatment option (9). The present case was characterized by a long segment (8 cm) benign anastomotic stenosis extending from the level of C7 to T5, which is refractory to endoscopic therapy. Therefore, we present a novel surgical approach including partial sternectomy, esophagectomy by a suprasternal approach, and a pedicled TAAP flap reconstruction with good result. A long stricture in the cervical and upper thoracic esophagus, which is in close proximity to the sternum, is indeed rare. Our approach to esophagectomy is an excellent option that is less invasive than transthoracic esophagectomy. In addition, based on the fact that the retrosternal route is commonly used in the postesophagectomy reconstruction process (10), we provide a good surgical option for patients with a secondary cervicothoracic esophageal lesion behind the sternum.

Generally, the choice of a reconstructive procedure for every defect depends on the location and length of the defect, availability of recipient vessels, selection of the reconstructive route, availability of donor organs and tissues, and general condition of the patient (11). For repairing partial hypopharyngeal defects with extension to the cervical esophagus, some pedicled flaps such as the pectoralis major myocutaneous flap, the latissimus dorsi myocutaneous flap, the platysma myocutaneous flap, and the sternocleidomastoid myocutaneous flap are reportedly used (12–14), with no specific pedicled or free flap being superior to others. For patients with circumferential hypopharyngectomy extending to the cervical esophagus, fasciocutaneous free flaps seem to represent the first-line option because of their low perioperative mortality, donor-site morbidity, and overall complication rates (5, 15). For those who undergo total thoracic or cervicothoracic esophagectomy when the lesions extend well below the thoracic inlet, the first choice for esophageal reconstruction is the use of the alimentary canal as a donor because it represents the replacement of “like for like” tissue (11, 16). Esophageal reconstruction with enteric flaps may be necessary in our patient, but this method is susceptible to perioperative mortality, donor-site-related complications, and flap failure, especially for a person with a poor health condition. For patients who undergo jejunum reconstruction, their perioperative mortality rates ranging from 0% to 17% (mean, 2.5%) are at least fourfold higher than those who reportedly use fasciocutaneous free flaps (5). Moreover, risk factors in our patient, such as smoking, radiation treatment, and refractory anastomotic fistula, may cause poor perfusion. Reliable recipient vessels are not always available when using fasciocutaneous free flaps. Pedicle flaps seem to be a better option, especially when considering the repair of a cervical anastomosis fistula for reasons described below. Thin pedicled flaps such as platysma myocutaneous flaps and sternocleidomastoid myocutaneous flaps are suitable for repairing short patch cervical esophageal defects (13, 17). Other pedicled flaps such as the pectoralis major myocutaneous flap and the latissimus dorsi myocutaneous flap are too bulky to be used in reconstructing esophageal stenosis. A pedicled TAAP flap is a good choice with advantages of stable blood supply, simple harvest process, and lower donor-site complications compared with microvascular free flaps or colonic interposition (18).

However, our technique has some limitations. The TAAP flap is not suitable for patients who have undergone previous surgery or for those who have been exposed to radiation on the ipsilateral chest region. Second, it is not suitable for female patients or those with abundant adipose tissue on the chest. Third, it is not suitable for repairing circumferential esophageal defects, because when the flap rolls up as a tubular conduit in such a narrow space, the risk of esophageal restenosis increases.

## Data Availability

The original contributions presented in the study are included in the article/Supplementary Material, further inquiries can be directed to the corresponding authors.
